# Exploring the Use of Personal Technology in Type 2 Diabetes Management Among Ethnic Minority Patients: Cross-Sectional Analysis of Survey Data from the Lifestyle Intervention for the Treatment of Diabetes Study (LIFT Diabetes)

**DOI:** 10.2196/diabetes.8934

**Published:** 2018-02-22

**Authors:** Sophie E Claudel, Alain G Bertoni

**Affiliations:** 1 School of Medicine Wake Forest University Winston Salem, NC United States; 2 National Heart, Lung, and Blood Institute National Institutes of Health Bethesda, MD United States; 3 Department of Epidemiology and Prevention Division of Public Health Sciences Wake Forest School of Medicine Winston Salem, NC United States; 4 Maya Angelou Center for Health Equity Winston Salem, NC United States

**Keywords:** diabetes mellitus, information technology, health disparities, race/ethnicity, continental population groups, ethnic groups, LIFT Diabetes Study

## Abstract

**Background:**

Minority populations have higher morbidity from chronic diseases and typically experience worse health outcomes. Internet technology may afford a low-cost method of ongoing chronic disease management to promote improved health outcomes among minority populations.

**Objective:**

The objective of our study was to assess the feasibility of capitalizing on the pervasive use of technology as a secondary means of delivering diabetic counseling though an investigation of correlates to technology use within the context of an ongoing diabetes intervention study.

**Methods:**

The Lifestyle Intervention for the Treatment of Diabetes study (LIFT Diabetes) randomly assigned 260 overweight and obese adults with type 2 diabetes mellitus to 2 intervention arms. At baseline, we administered a survey evaluating access to and use of various technologies and analyzed the responses using descriptive statistics and logistic regression.

**Results:**

The sample population had a mean age of 56 (SD 11) years; 67.3% (175/260) were female and 54.6% (n=142) self-identified as being from ethnic minority groups (n=125, 88.0% black; n=6, 4.3% Hispanic; and n=11, 7.7% other). Minority participants had higher baseline mean body mass index (*P*=.002) and hemoglobin A_1c_ levels (*P*=.003). Minority participants were less likely to have a home computer (106/142, 74.7% vs 110/118, 93.2%; *P*<.001) and less likely to have email access at home (*P*=.03). Ownership of a home computer was correlated to higher income (*P*<.001), higher educational attainment (*P*<.001), full-time employment (*P*=.01), and ownership of a smartphone (*P*=.001). Willingness to complete questionnaires online was correlated to higher income (*P*=.001), higher education (*P*<.001), full-time employment (*P*=.01), and home access to a computer, internet, and smartphone (*P*≤.05). Racial disparities in having a home computer persisted after controlling for demographic variables and owning a smartphone (adjusted OR 0.26, 95% CI 0.10-0.67; *P*=.01). Willingness to complete questionnaires online was driven by ownership of a home computer (adjusted OR 3.87, 95% CI 1.14-13.2; *P*=.03).

**Conclusions:**

Adults who self-identified as being part of a minority group were more likely to report limited access to technology than were white adults. As ownership of a home computer is central to a willingness to use online tools, racial disparities in access may limit the potential of Web-based interventions to reach this population.

**Trial Registration:**

ClinicalTrials.gov NCT01806727; https://clinicaltrials.gov/ct2/show/NCT01806727 (Archived by WebCite at http://www.webcitation.org/6xOq2b7Tv)

## Introduction

Approximately 92% of US adults own a mobile phone and 73% own a computer; therefore, it is critical to understand technology’s role in effective health care delivery, particularly if the convenience of personal technology can further the goals of decreasing health care costs while promoting improved health outcomes [1,2]. Type 2 diabetes mellitus is an increasingly common chronic disease that carries substantial health care costs and places a significant personal burden on patients to sustain adequate management. The potential for a convenient technology-based intervention is, therefore, especially relevant to diabetes management, as avoidable and costly complications of neuropathy, nephropathy, and retinopathy have debilitating and irreversible impacts on patients’ quality of life [3]. Recent, preliminary data suggest that using mobile phones, telecommunication, or email messaging with certified health coaches can facilitate significant weight loss, increase physical activity, and decrease hemoglobin A_1c_ (HbA_1c_) levels [4-6]. Pludwinski et al reported that smartphone-based resources facilitated decreasing HbA_1c_ levels in ethnic minority patients of low socioeconomic status by effectively strengthening the therapeutic alliance between patient and health coach [7], findings supported by other authors [8-10]. Furthermore, several studies documented a marked increase in patients’ self-efficacy, knowledge, and social support in addition to a reduction in cognitive load [7,8,11-14]. While these results are encouraging, patients’ ability to access these technologies among a more diverse population must be studied to avoid the futile outcome of developing an underused technology [13,14]. In this study, we sought to assess the feasibility of capitalizing on the pervasive use of technology as a secondary means of delivering diabetic counseling though an investigation of correlates to technology use within the context of an ongoing population health study. It is unclear whether study participants have sufficient access to the technologies that would support a translational intervention. We hypothesized that this investigation of an untapped resource in diabetes care among a high-risk population might be able to uncover the potential to support a novel, low-cost solution to a significant public health challenge facing the United States.

## Methods

### LIFT Diabetes Study Design

Overweight and obese adults with type 2 diabetes mellitus (N=260) were recruited from Forsyth County, NC, USA to participate in a study of lifestyle modifications for effective risk factor control and the prevention of disease complications—the Lifestyle Intervention for the Treatment of Diabetes study (LIFT Diabetes). Participants were recruited between 2013 and 2015 using a variety of approaches, including mailing potentially eligible adults identified in the electronic medical records system, direct referrals (primarily from health providers), advertisements, and community health events. Details regarding recruitment have been published [15]. The participants, primarily low-income and minority patients, were randomly assigned to 1 of 2 diabetes education groups: 1 consisted of a weekly intensive group-based lifestyle intervention promoting weight loss in a community setting and the other consisted of monthly diabetes self-management education resources delivered in the clinical setting. A more detailed description of the LIFT Diabetes protocol and design can be found in Katula et al [16]. At the baseline visit, all participants completed a survey, which had been used previously in the Action for Health in Diabetes (Look AHEAD) study (which also involved a lifestyle intervention for adults with diabetes) [17]. This technology use survey posed participants multiple questions regarding their current access to specific technologies, use of specific functions, and frequency of use. Additionally, the survey evaluated how participants would prefer to receive study information and whether they would be willing to complete future questionnaires online. The study reported here is an analysis of participants’ responses to this survey. The study design, methodology, and data collection protocols were approved by the Wake Forest Institutional Review Board, and written informed consent was obtained from participants. We handled all deidentified data in the statistical package Stata/IC version 14.1 (StataCorp LLC).

### Statistical Analysis

We compared frequencies and means of demographic characteristics, health outcomes, and survey responses through Pearson chi-square or Fisher exact analysis and *t* tests. To understand the racial disparities within the sample, we stratified overall demographic characteristics by racial/ethnic group. Due to the small number of nonblack minorities (n=17), as well as their similarity in health and demographic trends to black participants, we grouped all minority participants together for analysis. Due to the assumption that a technology-based intervention would require participants to engage with a device at least once a week, we collapsed all survey responses indicating frequency of use into 2 groups (at least once a week vs less than once a week). Following a description of the sample means and demographics, we used logistic regression as a means of understanding the relative impact of each demographic characteristic on survey responses. To limit the number of missing values in the regressions, questions that participants were prompted to skip after answering “no” to the previous question were recoded as “no” rather than “missing.” For example, if a participant did not own a mobile phone, in the subsequent question regarding smartphone ownership, their answer was coded as “no” rather than “missing.” Outcome variables used were ownership of a computer and willingness to complete future study questionnaires online.

## Results

### Data Set and Sample

We collected demographic information, health characteristics, and survey responses from each of the 260 LIFT Diabetes participants; therefore, we included all study participants in the descriptive statistics. However, due to missing information regarding employment, smartphone ownership, and text messaging, the final regression sample for ownership of a computer consisted of 257 participants, and the regression sample for willingness to complete future surveys online consisted of 208 participants.

### Descriptive Statistics

Table 1 shows the sample population’s demographic and baseline health characteristics. Of the ethnic minority participants, 88.0% were black (125/142), 4.3% were Hispanic (n=6), and 7.7% were other (n=11). Although 22.3% (58/260) of the study population declined to report annual income, most of the respondents (61.4%) reported an annual income of US $49,999 or less. Minority participants were much more likely to have a lower income than white participants (*P*<.001). Education followed similar, though not significant, trends between racial/ethnic groups, with minority participants typically achieving lower educational attainment. Overall, however, most of the study population (n=207, 79.6%) achieved greater than high school education. Of the population health characteristics, we found significant racial/ethnic differences in mean age (*P*<.001), body mass index (BMI; *P*=.002), diastolic blood pressure (*P*<.001), and HbA_1c_ (*P*=.003), suggesting a slightly worse baseline health profile among minority participants.

Table 2 shows technology access and use variables. Minority participants were significantly less likely to own a home computer (*P*<.001) and have email access at home (*P*=.03). To assess the participants’ ability and willingness to engage in future technology-based interventions, we correlated survey responses to demographic, health, and access variables (Table 3). Those who owned a home computer were more likely to have a higher income (*P*<.001), have higher educational attainment (*P*<.001), be employed full time (*P*=.01), and own a smartphone (*P*=.001). As expected, those with higher education and income were also significantly more likely to indicate a willingness to participate in technology-based surveys (*P* ≤.001). Similarly, those who were not employed full time were less likely to indicate a willingness to complete future study questionnaires online (*P=*.01). Each of the access variables also played a significant role in participants’ responses (*P* ≤.05). Of those who were not willing to complete online questionnaires, 52% (17/33) owned a home computer and 46% (15/33) had access to the internet at home.

### Logistic Regression

Table 4 presents the results of the regression analysis for owning a home computer and willingness to complete questionnaires online, when adjusted for demographic characteristics (age, sex, race/ethnicity, education, employment, family size, marital status) and health status (duration of diabetes, smoking status). Minority participants had significantly lower odds of owning a home computer (adjusted OR 0.26, 95% CI 0.10-0.67; *P=*.01). Those with a high school education or General Education Development also had lower odds of owning a home computer (adjusted OR 0.25, 95% CI 0.10-0.60; *P=*.002), while those who owned a smartphone had 3.11 higher odds of owning a computer (adjusted OR 3.11, 95% CI 1.37-7.08; *P*=.01). Education also played a role in willingness to complete questionnaires online, as those with a below–high school education had significantly lower odds of responding “yes” (adjusted OR 0.45, 95% CI 0.01-0.34; *P*=.003). Additionally, those who owned a home computer were more likely to be willing to complete questionnaires online (adjusted OR 3.87, 95% CI 1.14-13.2; *P=*.03).

**Table 1:** Demographic and health characteristics.

Characteristics		All participants (N=260)	Minority participants (n=142)	White participants (n=118)	*P* value
**Sex, n (%)**					.002
	Male	85 (32.7)	35 (24.7)	50 (42.4)	
	Female	175 (67.3)	107 (75.4)	68 (57.6)	
Age (years), mean (SD)		56 (11)	53 (11)	59 (10)	<.001
**Income (US $), n (%)**					<.001
	0-29,999	78 (30.0)	59 (41.6)	19 (16.1)	
	30,000-49,999	46 (17.7)	25 (17.6)	21 (17.8)	
	≥50,000	78 (30.0)	22 (15.5)	56 (47.5)	
	Missing or declined to answer	58 (22.3)	36 (25.4)	22 (18.6)	
**Educational attainment, n (%)**					.08
	Less than high school	9 (3.5)	8 (5.6)	1 (0.9)	
	High school/GED^a^	44 (16.9)	26 (18.3)	18 (15.3)	
	Greater than high school	207 (79.6)	108 (76.1)	99 (83.9)	
Employed, n (%)		216 (83.4)	115 (81.6)	101 (85.6)	.39
Insured, n (%)		235 (90.4)	125 (88.0)	110 (93.2)	.16
**Marital status, n (%)**					<.001
	Never married	47 (18.1)	39 (27.5)	8 (6.8)	
	Previously married	72 (27.7)	39 (27.5)	33 (28.0)	
	Married or equivalent	141 (54.2)	64 (45.1)	77 (65.3)	
**Family size (no. of persons), n (%)**					.44
	0-1	56 (21.5)	29 (20.4)	27 (22.9)	
	2-4	185 (71.2)	100 (70.4)	85 (72.0)	
	≥5	19 (7.3)	13 (9.2)	6 (5.1)	
**Smoking status, n (%)**					<.001
	Current smoker	40 (15.4)	26 (18.3)	14 (11.9)	
	Former smoker	86 (33.1)	32 (22.5)	54 (45.8)	
	Nonsmoker	134 (51.5)	84 (59.2)	50 (42.4)	
**Weight category, n (%)**					.18
	Overweight (BMI^b^ <30 kg/m^2^)	40 (15.4)	18 (12.7)	22 (18.6)	
	Obese (BMI ≥30 kg/m^2^)	220 (84.6)	124 (87.3)	96 (81.4)	
BMI (kg/m^2^), mean (SD)		37 (8)	39 (9)	36 (7)	.002
Waist circumference (cm), mean (SD)		120 (19)	122 (22)	118 (15)	.15
Systolic blood pressure (mmHg), mean (SD)		125 (15)	127 (16)	124 (15)	.09
Diastolic blood pressure (mmHg), mean (SD)		76 (10)	78 (10)	74 (10)	<.001
Triglycerides (mg/dL), mean (SD)		147 (100)	126 (80)	173 (115)	<.001
Hemoglobin A_1c_ (%), mean (SD)		7.6 (1.3)	7.8 (1.4)	7.3 (1.2)	.003
Fasting glucose (mg/dL), mean (SD)		149 (54)	148 (59)	150 (47)	.76
Diabetes duration (years), mean (SD)		8 (8)	9 (8)	8 (7)	.43
**Study arm, n (%)**					.21
	Lifestyle weight loss	130 (50.0)	76 (53.5)	54 (45.8)	
	Diabetes self-management	130 (50.0)	66 (46.5)	64 (54.2)	

^a^GED: General Education Development.

^b^BMI: body mass index.

**Table 2:** Technology use profile.

Survey questions		All participants (N=260), n (%)	Minority participants (n=142), n (%)	White participants (n=118), n (%)	*P* value
Own a home computer		216 (83.1)	106 (74.7)	110 (93.2)	<.001
Have email on home computer		206 (94.9)	98 (91.6)	108 (98.2)	.03
Check email on home computer at least once a week		117 (89.3)	60 (88.2)	57 (90.5)	.68
Have internet access on home computer		212 (97.7)	104 (97.2)	108 (98.2)	.68
Use the internet at home at least once a week		177 (84.7)	85 (82.5)	92 (86.8)	.39
Use the internet outside of home		185 (71.4)	95 (66.9)	90 (76.9)	.08
Use the internet outside of home at least once a week		155 (84.2)	80 (85.1)	75 (83.3)	.74
**Locations of non–home internet use**					
	Cyber café	12 (4.6)	5 (3.5)	7 (5.9)	.36
	Library	31 (11.9)	24 (16.9)	7 (5.9)	.01
	Family/friend’s home	48 (18.5)	23 (16.2)	25 (21.2)	.30
	Work	98 (37.7)	51 (35.9)	47 (39.8)	.52
	Other location	63 (24.2)	28 (19.7)	35 (29.7)	.06
Own a mobile phone		245 (94.2)	135 (95.1)	110 (93.2)	.52
Own a smartphone		161 (66.2)	86 (64.7)	75 (68.2)	.56
Can send and receive text messages on mobile phone		210 (86.8)	118 (87.4)	92 (86.0)	.75
Can send and receive emails on mobile phone		132 (54.6)	69 (51.1)	63 (58.9)	.23
Send or receive text messages at least once a week		197 (94.3)	111 (94.9)	86 (93.5)	.67
Use email on mobile phone at least once a week		117 (89.3)	60 (88.3)	57 (90.5)	.68
Use social networking		173 (66.5)	89 (62.7)	84 (71.2)	.15
**Use social networking at least once a week**					
	Facebook	134 (79.3)	67 (47.2)	67 (56.8)	.45
	Twitter	15 (10.6)	8 (5.6)	7 (5.9)	.79
	Skype	13 (9.2)	3 (2.1)	10 (8.5)	.08
	Other	18 (17.8)	9 (6.3)	9 (7.6)	.68
**Preferred method of contact**					
	Home phone	74 (28.5)	42 (29.6)	32 (27.1)	.66
	Mobile phone	150 (57.7)	88 (62.0)	62 (52.5)	.13
	Text message	93 (35.8)	54 (38.0)	39 (33.1)	.40
	Email	157 (60.4)	73 (51.4)	84 (71.2)	<.001
	US mail	99 (38.1)	64 (45.1)	35 (29.7)	.01
Would complete future online study questionnaires		227 (87.3)	121 (85.2)	106 (89.8)	.27

**Table 3:** Survey responses by demographic and health characteristics.

Characteristics		Own a home computer (n=216)		Willing to complete questionnaires online (n=227)	
		n (%)	*P* trend^a^	n (%)	*P* trend
**Age group (years)**			.22		.63
	<65	163 (75.5)		175 (77.1)	
	≥65	53 (24.5)		52 (22.9)	
**Sex**			.63		.63
	Female	144 (66.7)		154 (67.8)	
	Male	72 (33.3)		73 (32.2)	
**Income (US $)**			<.001		.001
	0-29,999	53 (24.5)		61 (26.9)	
	30,000-49,999	40 (18.5)		43 (18.9)	
	≥50,000	75 (34.7)		75 (33.0)	
	Missing or declined to answer	48 (22.2)		48 (21.2)	
**Educational attainment**			<.001		<.001
	Less than high school	5 (2.31)		4 (1.8)	
	High school/GED^b^	28 (13.0)		34 (15.0)	
	Greater than high school	183 (84.7)		189 (83.3)	
Employed full time		104 (48.2)	.01	108 (47.6)	.01
**Weight category**			.72		.97
	Overweight (BMI^c^ <30 kg/m^2^)	34 (15.7)		35 (15.4)	
	Obese (BMI ≥30 kg/m^2^)	182 (84.3)		192 (84.6)	
**Glycemic control (hemoglobin A_1c_)**			.71		.36
	Good control (≤7.0%)	90 (41.7)		91 (40.1)	
	Poor control (7.0%)	126 (58.3)		136 (60.0)	
Diagnosed hypertension		193 (89.4)	.51	204 (89.9)	.76
Own home computer		N/A^d^	N/A	199 (87.7)	<.001
Have internet access at home		211 (97.7)	.98	197 (98.5)	.05
Use the internet at home at least once a week		176 (84.6)	.85	169 (74.5)	<.001
Own a smartphone		144 (70.9)	.001	149 (69.0)	.01

^a^*P* values determined by chi-square, Fisher exact, or *t* test.

^b^GED: General Education Development.

^c^BMI: body mass index.

^d^N/A: not applicable.

**Table 4:** Fully adjusted regression results^a^.

Characteristics		Own a home computer (n=257)		Willing to complete questionnaires online (n=224)	
		OR (95% CI)	*P* value	OR (95% CI)	*P* value
Age		1.03 (0.99-1.08)	.17	0.98 (0.93-1.04)	.56
Male		1.30 (0.51-3.29)	.58	1.52 (0.49-4.71)	.47
Minority		0.26 (0.10-0.67)	.01	1.29 (0.43-3.86)	.65
**Education**					
	Less than high school	0.22 (0.04-1.18)	.08	0.45 (0.01-0.34)	.003
	High school/GED^b^	0.25 (0.10-0.60)	.002	0.52 (0.17-1.62)	.26
	Greater than high School	reference		reference	
Employed full time		2.33 (0.95-5.69)	.06	1.67 (0.59-4.77)	.34
Student		9.72 (0.93-100.9)	.06	N/A^c^	N/A
**Family size (no. of persons)**					
	0-1	reference		N/A	N/A
	2-4	1.85 (0.68-5.03)	.23	N/A	N/A
	≥5	1.94 (0.42-9.05)	.40	N/A	N/A
**Marital status**					
	Never married	reference		N/A	N/A
	Previously married	0.99 (0.33-2.96)	.98	N/A	N/A
	Married or equivalent	1.21 (0.38-3.83)	.75	N/A	N/A
Diabetes duration		0.98 (0.93-1.03)	.42	N/A	N/A
Current or former smoker		1.42 (0.63-3.19)	.39	N/A	N/A
Own a smartphone		3.11 (1.37-7.08)	.01	1.48 (0.52-4.17)	.46
Own a home computer		N/A	N/A	3.87 (1.14-13.2)	.03
Use social networking		N/A	N/A	1.87 (0.62-5.66)	.27
Send or receive text messages at least once a week		N/A	N/A	1.08 (0.78-1.49)	.65

^a^Model is adjusted for demographic characteristics (age, sex, race/ethnicity, education, employment, family size, marital status) and health status (diabetes duration, smoking status).

^b^GED: General Education Development.

^c^N/A: not applicable.

## Discussion

### Principal Findings

The objective of this study was to determine both access to and use of technology among an ethnic minority population to assess the correlates to use of information technology among adults enrolled in a diabetes study. Broadly, at proportions consistent with national averages, most of the sample owned a home computer and mobile phone and had access to email and the internet [1]. However, significant dissimilarity between racial/ethnic groups was evident in demographics, health, and technology access. The racial disparity in income was reflected in the strong correlation between income and both ownership of a home computer and willingness to complete questionnaires online. The associated racial disparity in owning a home computer persisted even after controlling for numerous demographic characteristics, suggesting that socioeconomic status cannot fully explain the digital divide along racial lines. Subsequent analysis demonstrated that willingness to complete questionnaires online depends heavily on home computer access, which parallels a recent finding that home internet access drives patients’ willingness to use technology for glycemic monitoring [18]. Together, these results indicate that access to home technology is critical to the advancement of Web-based interventions, yet a significant racial discrepancy in access limits practical, translational implementation among a minority population. Regardless of literature citing effectiveness, the potential success of a technology-based program is irrelevant without sufficient access [5,7,8]. Yet the results also indicate that access alone may not be the sole barrier to such an intervention. Approximately 50% of the study sample indicated that they had access to both a home computer and the internet yet indicated “no” when asked about completing study questionnaires online. While it is perhaps surprising that those with home access to technology would indicate an unwillingness to participate in a technology-based intervention, these findings suggest that, while access is a critical determinant for any future intervention, purely supplying resources may not be sufficient. Whether this is an issue of familiarity with using technology or a deeper discomfort with nontraditional clinical settings, it is evident that initiating a Web-based protocol among minority patients with diabetes may fail to capture a meaningful portion of the population.

The significant relationship between educational attainment and an interest in online platforms presented here parallels previous discussions of education and telecommunication [18,19]. While higher education typically correlates to higher income, concurrent relatively low income and relatively high education seen in this study may not be unrealistic. Dray-Spira et al suggested that diabetes may impair patients’ ability to maintain the standard of employment associated with higher education, or that diabetes-related disability results in significant work loss followed by termination of employment at higher levels [20]. These possibilities are bolstered by the low level of full-time employment in this study (116/260, 44.6%) and broader consideration that work disability days are significantly higher for employees with diabetes [21,22]. Therefore, significant diabetes-related work disability may have exacerbated the disparity in education status and income seen in the study population.

Although health characteristics are unlikely to influence willingness to participate in Web-based programs, it is possible that underlying health problems could influence earning capacity and thus also access to technology. However, the health technology access and health technology use correlations explored were not statistically significant (data not shown). Despite this negative finding, the health metrics described reinforce previously observed racial disparities in health outcomes [23,24]. Prior research demonstrated that these disparities cannot be adequately accounted for by childhood socioeconomic status, adult income disparities, or health behaviors, but rather by the influence of allostatic load, exposure to discrimination, and decreased social capital [24,25]. While we did not explore these social variables in this study, we did find the expected trend that minority participants with diabetes exhibited higher BMI, diastolic blood pressure, and HbA_1c_ than did white participants. Therefore, while the sample was geographically restricted, the observed health patterns are generally consistent with other research studies.

### Study Limitations

This investigation was limited by its reliance on a single survey item—willingness to complete questionnaires online—as a proxy for participants’ willingness to engage in health coaching or health metric tracking through Web-based technology. A more robust analysis would be facilitated by additional, specific questions focusing on the issue of telecommunication in diabetes management. Demographically, the study had relatively few black men and generally had few nonblack minority participants, which necessitated grouping these participants into a general minority classification, and this therefore may have obscured distinct minority group responses. Finally, the study was restricted to a population of patients with diabetes already under a physician’s care at the time of enrollment and who were willing to be randomly assigned to a clinical trial, which may indicate higher socioeconomic position and therefore limits the generalizability of the findings to a broader minority population.

### Conclusions

This study established demographic characteristics, health profiles, and access to technology within an ethnic minority population in the southeastern United States. Research on chronic disease management—specifically diabetes—and clinical practice have demonstrated the effectiveness of intensive behavioral and lifestyle interventions in reducing the risk of disease complications [26-28]. Smartphone apps, telecommunication, and other mobile technologies have been proposed as efficient and effective alternatives [4,29-31]. However, these findings have yet to be reconciled with the results presented above—that minority patients of lower socioeconomic status lack both access to and familiarity with certain computer technologies—which limits the possibility for a translational intervention. While advancing diabetes research to address health disparities will require an innovative approach, the argument for mobile technology is not well supported at this time. In addition to focusing intervention efforts in other areas, studying minority patients’ perceptions of technology in the clinical setting may provide a much-needed perspective and inform the use of Web-based apps when technology becomes a viable approach. Here we highlighted the limited feasibility of introducing mobile technology to reduce health disparities among those with diabetes. However, as access to technology increases with time, future studies should investigate users’ perceptions of data safety and privacy, the cost of data plans associated with mHealth tools, and barriers to using personal technology in the clinical setting, aside from resource deprivation.

## Abbreviations

BMI: body mass index
GED: General Education Development
HbA_1c_: hemoglobin A_1c_
LIFT Diabetes: Lifestyle Intervention for the Treatment of Diabetes study
Look AHEAD: Action for Health in Diabetes
